# Extracting robust single-trial somatosensory evoked potentials for non-invasive brain computer interfaces

**DOI:** 10.1088/1741-2552/adfd8a

**Published:** 2025-09-03

**Authors:** Disha Gupta, Jodi Brangaccio, Helia Mojtabavi, Jonathan Wolpaw, N Jeremy Hill

**Affiliations:** 1 National Center for Adaptive Neurotechnology, Stratton Veterans Affairs Medical Center, Albany, NY 12208, United States of America; 2 Electrical and Computer Engineering, University of Albany, State University of NY, Albany, NY, United States of America

**Keywords:** somatosensory evoked potentials, brain computer interfacing, single-trial decoding, tibial nerve stimulation

## Abstract

*Objective.* Reliable extraction of single-trial somatosensory evoked potentials (SEPs) is essential for developing brain-computer interface (BCI) applications to support rehabilitation after brain injury. For real-time feedback, these responses must be extracted prospectively on every trial, with minimal post-processing and artifact correction. However, noninvasive SEPs elicited by electrical stimulation at recommended parameter settings (0.1–0.2 msec pulse width, stimulation at or below motor threshold, 2–5 Hz frequency) are typically small and variable, often requiring averaging across multiple trials or extensive processing. Here, we describe and evaluate ways to optimize the stimulation setup to enhance the signal-to-noise ratio (SNR) of noninvasive single-trial SEPs, enabling more reliable extraction. *Approach.* SEPs were recorded with scalp electroencephalography in tibial nerve stimulation in thirteen healthy people, and two people with CNS injuries. Three stimulation frequencies (lower than recommended: 0.2 Hz, 1 Hz, 2 Hz) with a pulse width longer than recommended (1 msec), at a stimulation intensity based on H-reflex and M-wave at Soleus muscle were evaluated. Detectability of single-trial SEPs relative to background noise was tested offline and in a pseudo-online analysis, followed by a real-time demonstration. *Main* *results.* SEP N70 was observed predominantly at the central scalp regions. Online decoding performance was significantly higher with Laplacian filter. Generalization performance showed an expected degradation, at all frequencies, with an average decrease of 5.9% (multivariate) and 6.5% (univariate), with an AUC score ranging from 0.78–0.90. The difference across stimulation frequencies was not significant. In individuals with injuries, AUC of 0.86 (incomplete spinal cord injury) and 0.81 (stroke) was feasible. Real-time demonstration showed SEP detection with AUC of 0.89. *Significance.* This study describes and evaluates a system for extracting single-trial SEPs in real-time, suitable for a BCI-based operant conditioning. It enhances SNR of individual SEPs by alternate electrical stimulation parameters, dry headset, and optimized signal processing.

## 1. Introduction

Brain and spinal injury lead to debilitating sensory and motor impairments that affect quality of life (Ozdemir and Perez 2018, Peterson *et al* 2019, Lo *et al* 2021). Much research is focused on rehabilitating the motor connections for active movement execution, and less attention is given to improving afferent connections for somatosensory feedback from the limbs (Hendricks *et al* 2002, Langhorne *et al* 2009, Zandvliet *et al* 2020). Somatosensory feedback is known to be important for motor processes (Gale *et al* 2021). Plasticity is possible in these pathways (Jones and Pons 1998, Chand and Jain 2015), and observed to be associated with functional improvements (Bolognini *et al* 2016, Turville *et al* 2017), making it a potential target for rehabilitation interventions. Recovery of the evoked responses have been observed to be associated with functional recovery (Rowed *et al* 1978, Pan *et al* 2017), and interventions based on external modulation (such as operant conditioning (Skinner 1938)) of evoked responses (cortical: Fox and Rudell 1968, Rosenfeld *et al* 1969, spinal: Wolpaw and Dowman 1988) have also shown potential for functional recovery (Finley 1984, Dowman and Rosenfeld 1985a, Dowman and Rosenfeld 1985b, Miltner *et al* 1988, Thompson *et al* 2009, 2013, Thompson and Wolpaw 2021).

In an Operant Conditioning protocol, for a specific stimulus, a desired response is rewarded. After repeated exposures, the response occurs more often (Skinner 1938). Several studies have used this protocol to show that humans and animals can gradually change the size of an evoked potential, such as the spinal stretch reflex (the sensory responses that travel from sensory endings in the muscle spindles to the spinal cord), or its electrical analog the H-reflex, when rewarded for doing so (Wolpaw *et al* 1983, Wolpaw 1987, Evatt *et al* 1989, Thompson *et al* 2009, 2013, Thompson and Wolpaw 2021). This protocol affects the spinal pathway itself; affecting several behaviors that use that pathway, enabling effective practice and thereby functional improvements, such as symmetrical walking and increased walking speed (Thompson *et al* 2013, Thompson and Wolpaw 2021). The operant conditioning of cortical somatosensory evoked potentials (SEPs)—widely used to examine the integrity of the afferent pathways (Chiappa and Ropper 1982, Chiappa 1997, Chabot *et al* 1985, Schaefer *et al* 2002, Poornima *et al* 2013), and known to be diminished/delayed post injury (Chabot *et al* 1985, Zileli *et al* 1991, Curt and Dietz 1996, Ma *et al* 2011, Cui *et al* 2015, Gupta *et al* 2017, Ozdemir and Perez 2018, Peterson *et al* 2019)—within such a closed-loop targeted neuroplasticity framework has been much less studied (Finley 1984, Dowman and Rosenfeld 1985a, 1985b, Miltner *et al* 1988). A cortical operant conditioning system would ideally deliver closely paired feedback for every evoked response, in real time. We outline key challenges in designing this system and evaluate potential solutions.

*Extracting single SEPs:* invasive SEP recordings obtained directly from the cortical surface—such as via electrocorticography, can typically yield individual SEPs with high signal-to-noise ratio due to their proximity to the neural source (Rettore Andreis *et al* 2024). In contrast, noninvasive SEP recordings from the scalp are generally attenuated, noisier and more challenging to differentiate from background noise, both within and across recording sessions (Cecotti and Ries 2017). As a result, these signals were traditionally extracted by averaging across hundreds of trials (Childers *et al* 1987, Allison *et al* 1992, Misulis and Spehlmann 1994, Delorme and Makeig 2004, Luck 2014 over several minutes of data; Mouraux and Iannetti 2008), followed by the analysis of peak amplitudes and latencies of characteristic components of the averaged response. This approach assumes that evoked responses are reliably repeatable and precisely time-locked to stimulus onset across all trials. However, studies highlight the nonstationary nature of EEG signals, with trial-to-trial variations in magnitude, topography, and latency, due to prior brain states and ongoing neural processes (Kutas *et al* 1977, Yabe *et al* 1993, Haig *et al* 1995, Jongsma *et al* 2000, Makeig *et al* 2002, Changoluisa *et al* 2020), attention (Hillyard *et al* 1973), and repetition suppression (Quiroga *et al* 2007)—fatigue, sharpening, or facilitation (Merchie and Gomot 2023). In individuals with brain or spinal injuries, additional variability can arise from post-injury anatomical (altered and disturbed conduction and dispersion due to axonal and nerve loss) and cognitive sequelae, sensory inattention at the disused limb, pain, and muscle spasticity or fatigue (Cui *et al* 2015). Moreover, issues such as movement, electrode pop, sweat, or an electrical artifact can also affect the magnitude of some of the responses.

In online BCI approaches, methods have evolved to provide results from a few seconds of the signal, but most ERP-based BCIs still rely on averaging responses across 10–15 stimulus presentations within those few seconds to mitigate the effects of background noise and obtain a usable result (Wolpaw *et al* 2002, Singh *et al* 2021). They also benefit from contrasting multiple stimulus-driven conditions, thereby allowing the classifier to focus on the differences between brain responses, with the common noise subtracted out (For example, left vs. right, target vs. non-target) (Wolpaw *et al* 2002, Hill *et al* 2012, Cecotti and Ries 2017). However, an operant conditioning setup demands even-more granular analysis so that feedback can be given after each individual response; so, the system faces the challenge of detecting *single* ERPs in the presence of background noise. This is rarely done. A few studies have demonstrated that it is possible in certain domains, as in the exploitation of error-related negativity (Chavarriaga *et al* 2014, Gomez-Andres *et al* 2024, Parashiva and Vinod 2021, Wirth *et al* 2019); but feasibility of online single-ERP extraction remains to be quantified in the somatosensory domain. The challenge is compounded in people with injuries, with an attenuated SEP response along with increased contamination, for example EEG noise due to drowsiness or fatigue, or EMG noise due to spasticity (Field-Fote *et al* 2022).

*Denoising and enhancing signal-to-noise-ratio:* The cortical conditioning system hence requires methods to reduce artifacts (such as due to movement or electrical noise) and enhance the SEP SNR in real time. Previous studies have demonstrated a range of advanced signal processing techniques for denoising and enhancing ERP SNR, such as Wavelet transform-based filtering (Quiroga and Garcia 2003, Ahmadi and Quiroga 2013, Amin *et al* 2023); phase space reconstruction methods (Effern *et al* 2000); independent component analysis (ICA) techniques (Jung *et al* 2001, Hu 2005, Rossi 2007, Liu *et al* 2011, Qin *et al* 2016, Lemm *et al* 2006); probabilistic ICA method (Hu *et al* 2011b); bayesian inference approaches (Truccolo *et al* 2003, Wu *et al* 2014); expectation maximization (Chen *et al* 2016); latency correction methods (Quiroga 2000); and PARAFAC or canonical decomposition methods (Vanderperren *et al* 2013, Miwakeichi *et al* 2004). Other methods that have been demonstrated for extracting single-trial ERPs for other sensory modalities, include multiple linear regression (Mayhew *et al* 2006), time sequence based adaptive filtering (Britton *et al* 2000), time frequency analysis with wavelet transforms (Mouraux and Plaghki 2004), generative networks (Zhang *et al* 2022), and a combination of wavelet filtering and multiple linear regression (Hu *et al* 2011). However, even-though, post-processing signal processing techniques that utilize noise covariance and trial-to-trial signal variability can improve the reliability and SNR of the evoked response (Blankertz *et al* 2011), a BCI application that needs to assess each individual response in real time, within milliseconds, limits the use of computationally intensive processes, complex denoising methods or the integration of retrospective cross-trial noise information.

*Proposed system:* borrowing elements from the H-reflex operant conditioning protocols (Wolpaw *et al*, Thompson and Wolpaw 2021) and previous studies (Gupta *et al* 2025), we propose a multi-pronged approach to enhance the SNR and detection accuracy of single-trial SEPs: (a) first, by optimizing stimulation parameters to evoke more consistent and distinct responses across trials and minimizing variability in effective afferent input through real-time monitoring and stabilization of the corresponding nerve and muscle response; (b) second, by reducing measurement noise by using an EEG acquisition system engineered for robustness against environmental, electrical, and movement-related artifacts; and (c) third, by improving SEP SNR and detection by optimizing the online SEP preprocessing, denoising, and feature extraction pipeline.

This involves: *first*, the use of stimulation parameters similar to an H-reflex operant conditioning protocol, with a longer pulse width of 1 msec to preferentially stimulate the sensory nerves; a higher stimulation intensity that elicits a measurable M-wave (Thompson *et al* 2009) that is 10%–20% of maximal M-wave and ∼75% of the maximum H-reflex (Binder *et al* 2009) at the innervated muscle, ensuring an afferent excitation that consistently reaches the deeper nerves from surface electrodes, and can be measured and maintained by observing a measurable consistent M-wave in real time; a lower frequency of 0.2–2 Hz to prevent excessive movement and attenuation of the SEP due to gating effects, that can occur due to the higher stimulation intensity; and maintaining a low level (5%–10% of maximum voluntary contraction) background EMG contraction in the soleus muscle to maintain balance and avoid post activation depression of the spinal afferent response. These stimulation parameters are different from those recommended for SEPs (Cruccu *et al* 2008)–0.1–0.2 msec pulse width, 2–5 Hz stimulation frequency and a stimulation intensity that is 2–3 times the sensory threshold (Brull and Silverman 1995) but have been found to be suitable to elicit robust SEPs without undue discomfort (Kato *et al* 2023, Gupta *et al* 2025). The Evoked Potential Operant Conditioning System (EPOCS) (Hill *et al* 2022) is used here, as it allows the real time measurement and monitoring of the H-reflex and M-wave suitable for this evaluation. *Second*, reduce the EEG contamination due to electrical and movement artifacts, expected to be larger at the proposed stimulation parameters, by using a dry active wireless EEG headset (such as the DSI-24, Wearable Sensing), with multiple hardware and software design features that can actively reduce such contamination. *Third*, evaluate various spatial filtering approaches followed by a comparison of multivariate versus univariate spatial features to optimize SEP detection accuracy.

We test the system described above in the context of non-invasive SEPs to tibial nerve stimulation at the popliteal fossa (Wang *et al* 2017), with the aim of assessing accurate detection of individual SEPs relative to ongoing background EEG fluctuations, in real time. We focus on the mid-latency somatosensory cortical response, the N70 (Eisen *et al* 1979, Avanzini *et al* 2018), which is known to reflect the lower-limb afferent signal transmitted via the dorsal column medial lemniscus pathway (Ebner *et al* 1982, Urasaki *et al* 1985). We evaluate the setup for generalization, to reduce the need for recalibration, and test-retest stability for longitudinal assessments. We conduct this evaluation under strict pseudo-online constraints in a cohort of healthy individuals, followed by an actual online demonstration in two healthy people. Since this BCI application is intended for practical use by individuals with CNS injuries-where the weakened or delayed SEPs (Chabot *et al* 1985, Kovala *et al* 1991, Fierro *et al* 1999, Zhang *et al* 2011) could further affect the single-trial detection–we perform a preliminary feasibility assessment in one individual with stroke and one with spinal cord injury.

## 2. Data and methods

**Participants:** thirteen healthy people participated in the study. *Exclusion criteria* included a history of neurological disease, use of neuromodulatory medications, open wounds or known scalp infections, pregnancy, or the presence of metal implants such as cardiac pacemakers, cochlear implants, neurostimulators, or metal rods. Two healthy participants displayed large shoulder movements in response to every stimulus. Their data was excluded from the analysis as it injected significant movement artifacts in the EEG. In addition to healthy participants, two people with CNS injuries also participated in this study. One individual had suffered a right brain ischemic stroke 15 years ago, with residual sensory and motor (plegia) impairments in left arm and left leg. The second individual had incomplete cervical spinal cord injury approx. One year ago; had marked sensory and motor impairments in both arms and legs and relied on a wheelchair for mobility. The research was conducted in accordance with the Declaration of Helsinki. All participants provided written informed consent, and the study protocol was approved by the local ethics institutional review board at Stratton VA Medical Center (#1584762).

**EEG Recording:** SEPs were recorded using a non-invasive referential EEG setup with a 19-channel EEG headset (DSI, Wearable Sensing, CA). The dry active electrodes were positioned according to the international 10-20 EEG system (Klem *et al* 1999) (Fp1, Fp2, Fz, F3, F4, F7, F8, Cz, C3, C4, T7/T3, T8/T4, Pz, P3, P4, P7/T5, P8/T6, O1, O2), with the ground electrode placed at FPz and the reference at linked earlobes. Data acquisition was performed at 300 Hz and with the BCI2000 software (Schalk *et al* 2004, Mellinger and Schalk 2007). To synchronize the EEG and EMG data, electrical stimulus pulses from the EPOCS system to the electrical stimulator were copied and transmitted to the EEG acquisition system as 5 V transistor-transistor logic (TTL) pulses.

**Peripheral Electrical Nerve Stimulation:** electrical stimulation was delivered using a constant current stimulator (DS8R, Digitimer Ltd) via surface electrodes placed on the tibial nerve at the popliteal fossa. A larger anode electrode (22 × 35 cm) was positioned at the apex of the popliteal fossa, while a smaller cathode electrode (22 × 22 cm) was placed 3 cm below the anode at the knee crease. Biphasic stimulation pulses of 1 ms (positive polarity) were used. Stimulation was applied at three frequencies (0.2 Hz, 1 Hz, and 2 Hz) with randomized 20% jitter, resulting in interstimulus intervals of 5 ± 1 s, 1 ± 0.2 s, and 0.5 ± 0.1 s, respectively. For each frequency, both Recruitment Curve and Assessment runs were collected.

*Recruitment curve run:* typically, recruitment curves for *H*-reflex and *M*-wave were first generated to determine the stimulation current that elicited the target compound muscle action potential (CMAP or *M*-wave) for subsequent assessment runs. These were generated by increasing the stimulation current amplitude steadily from below H-reflex threshold and M-wave threshold until the M-wave reaches an asymptote at its maximum value (*M*_max_). In an HrTNP protocol, these curves were used to identify the stimulation intensity that generated an *H*-reflex that was about 75% of *H*_max_ and has a visible *M*-wave (i.e. the target *M*-wave, typically 10%–20% of *M*_max_).

Assess*ment run:* next, for each participant, 75 trials were acquired at the above stimulation intensity, with small changes as needed in stimulation intensity to maintain the target *M*-wave. The background soleus EMG was maintained at a predefined low level sufficient for weight bearing when standing upright (typically 5%–10% of the maximum voluntary contraction).

**EMG recording:** bipolar EMG was recorded with an 8-channel signal acquisition system (AMT-8, Bortec Biomedical Ltd, Canada) that included a pre-amplifier with a gain of 500. Surface electrodes (Ag/AgCl, A10040-60, Vermont Medical Inc.) were positioned at the soleus muscle on the right leg, with the ground at patella, as described in Hill *et al* (Hill *et al* 2022) and Thompson *et al* (Thompson *et al* 2013, Thompson and Wolpaw 2021, McKinnon *et al* 2023). The data were bandpass filtered at 10–1000 Hz and digitized with an analog-to-digital convertor (PCIe-6321, National Instruments), at a sampling rate of 3200 Hz. The EMG data were continuously transmitted to the EPOCS computer in real-time. It monitored the background EMG and triggered the stimulator whenever background EMG was maintained at a pre-defined low-level for 2 sec in the 0.2 Hz, and 200 msec in the 1 and 2 Hz runs, while standing upright.

**Experimental protocol:** the experimental protocol evaluated the cortical SEPs evoked by tibial nerve stimulation at three frequencies, while maintaining a consistent *H*-reflex, background EMG, and *M*-wave size. Data was collected in a single session per participant, consisting of two blocks with six runs each. Each block included a Recruitment run followed by an Assessment run at each stimulation frequency. Recruitment runs determined the stimulation intensity needed to meet the HrTNP criterion as described above (i.e. an *H*-reflex ∼75% of *H*_max_ and an *M*-wave typically 10%–20% of *M*_max_ with stable background muscle contraction. Assessment runs collected 75 trials. The stimulation frequencies were randomized in the first block and reversed in the second block. Participants were able to sit and rest in between runs, as needed.

**Figure 1.**
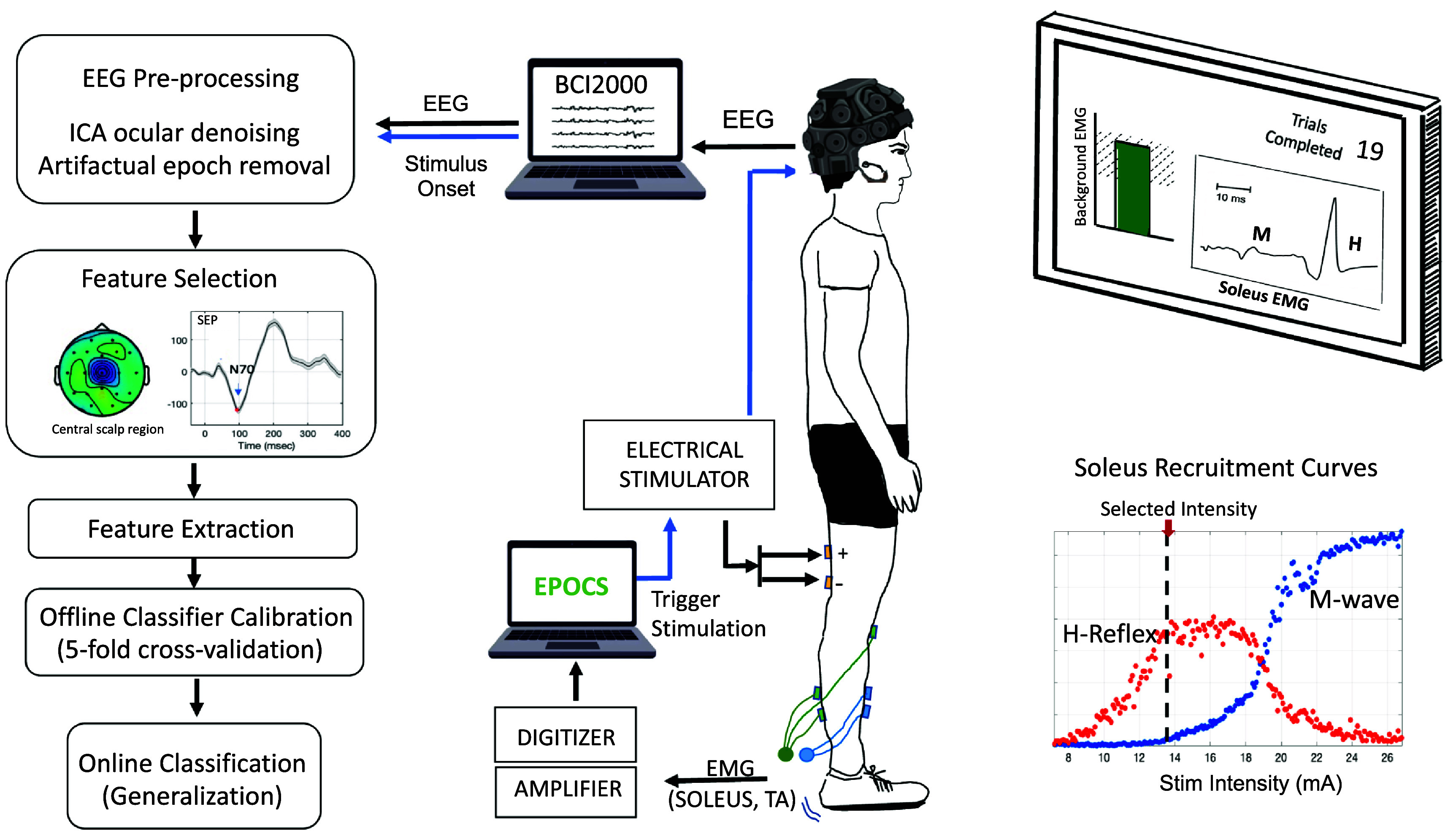
Experiment setup and analysis pipeline. The experiment setup includes tibial nerve stimulation with a constant current electrical stimulator, electromyography (EMG) acquisition system (analog amplifier, analog to digital convertor) to measure soleus EMG, electroencephalography (EEG) acquisition system (EEG headset, BCI2000 software (Schalk *et al* 2004)), our evoked potential operant conditioning system (EPOCS: (Hill *et al* 2022)). Stimulation and monitoring system, and a screen for real-time visualization of background EMG. The data plot inset shows the *H*-reflex and *M*-wave recruitment curves obtained in pseudo real-time, used to determine the stimulation intensity for the subsequent assessment run. Left panel shows the analysis pipeline used for assessing the performance of single-trial somatosensory evoked potential measurement.

The single-trial decoding analysis was performed in four main steps, as described here, and shown in figure 1.

*a)Data Pre-Processing:* the first step included denoising and feature selection.

*(i) Denoising:* the recruitment and assessment data runs were first denoised by identifying and removing bad channels, applying a band pass filter of 0.2–40 Hz (zero phase, Butterworth, model order 2). The assessment runs were not pre-processed any further, to mimic a real time SEP extraction. The recruitment runs were further processed with ICA to remove ocular artifacts (Jiang *et al* 2019), and trial statistics-based identification and removal of artifactual trials. (ii) *Feature Selection:* next, we used the recruitment curve data to estimate the subject-specific temporal feature i.e. the latency for N70 SEP responses, and to determine the most relevant spatial region-of-interest. Typically, for tibial nerve stimulation, the central scalp regions (Cz electrode) have been shown to be most active spatial regions (Penfield and Rasmussen 1950, Roux *et al* 2018). This region has also been associated with the lower limbs as per the cytoarchitectural and histological maps of the human brain (Brodmann area 3, 2, 1) (Garey 2006). For multivariate analysis, we use the electrode set that includes the Cz and its centro-parietal neighboring electrodes (C3, P3, Pz, C4, P4). For univariate analysis, we based the electrode selection on the coefficient-of-determination (*r*^2^), which reflects the signal-to-noise ratio and the spatial specificity of the scalp regions most active during the evoked response. As the assessment is binary, the labels used are arbitrary (−1, +1). b) *Feature Extraction:* Subsequent analysis was performed on Assessment data runs. Pre-processed data was epoched from a −25 to 25 ms window centered at the N70 or P40 latency obtained from the feature selection step a(ii). The background EEG epochs were created from 50 ms windows prior to the stimulation onset. These resting state epochs were obtained from −50 msec to 0 msec relative to each trial onset. A balanced dataset was created with an equal number of rest and SEP epochs. The RMS of these epochs, at the selected electrodes, were used as the features for the classification, guided by the broad temporal span and latency jitter observed in the N70 peak. c) *Offline Classifier Calibration:* the classification step involved an offline calibration phase that used only the first run, that included 75 trials per participant per stimulation frequency. The aim of this step was to quantify the specificity and sensitivity of eliciting SEPs distinguishable from background noise at every trial, and the stability of such responses across multiple sessions and unseen data. Here, we evaluate the effect of stimulation frequency (0.2 Hz, 1 Hz, 2 Hz) and the spatial filters (surface Laplacian (Perrin *et al* 1989), common average (McFarland *et al* 1997), and linked ears or bipolar), followed by the evaluation of multivariate classification using a feature set of six electrodes (C3, P3, Cz, Pz, P4, and C4), and testing the possibility of simplifying the procedure to univariate classification, based on a single spatial feature (Cz).

The classification was performed by extracting two data points per trial—one for SEP response and one from the pre stimulus resting state—and performing a regularized linear discriminant analysis (rLDA) (Fisher 1936). rLDA is a supervised machine learning algorithm that maximizes class separation by maximizing the ratio of between-class variance to within-class variance. The offline performance of the classifier was tested via a 5-fold cross-validation method (Stone 1974, Cecotti and Ries 2017), where the training was repeatedly performed on 4/5th of the dataset and tested on the remaining 1/5th of the data. Cross-validation is a model-based validation technique to assess how the classifier will generalize to unseen data. The classification performance was assessed with the area under the Receiver Operating Characteristics curve (ROC Area Under the Curve (AUC) score (Fawcett 2006)), useful in case of imbalanced class distribution as well (Cecotti and Ries 2017). AUC ranges from 0 to 1, where 0.5 indicates random guessing, and 1 is perfect performance. The overall AUC was calculated as the median across the five folds.

In the multivariate classification approach, the regularization for the rLDA was performed with a hyperparameter optimization step at each cross-validation fold, using a holdout test segment that was 20% of the cross-validation fold data.

We also used the training data to retrospectively assess the spatial distribution of the SEP N70 response across the electrodes. Its signal-to-noise ratio and the spatial specificity was assessed with *r*^2^. The significance of *r*^2^ was measured across all electrodes as well, and the Bonferroni corrected −log_10_(*p*) values were plotted on the topography for ease of visual interpretation. d) *Pseudo-Online Classification:* this step applied the learnt classifier to unseen test data to assess single-trial generalization. For this step, we used the second assessment run at each stimulation frequency (i.e. 75 trials per participant). The data de-noising steps were kept minimal (i.e. linear and spatial filtering only) to mimic the online data pre-processing pipeline in a typical online BCI setup, such that the pre-processing did not need to see all the trials as is required in ICA based denoising and statistics based artifactual trial removal. The spatial filters were based on prior calibration data. The classification performance was measured by the ROC AUC scores.

**Figure 2.**
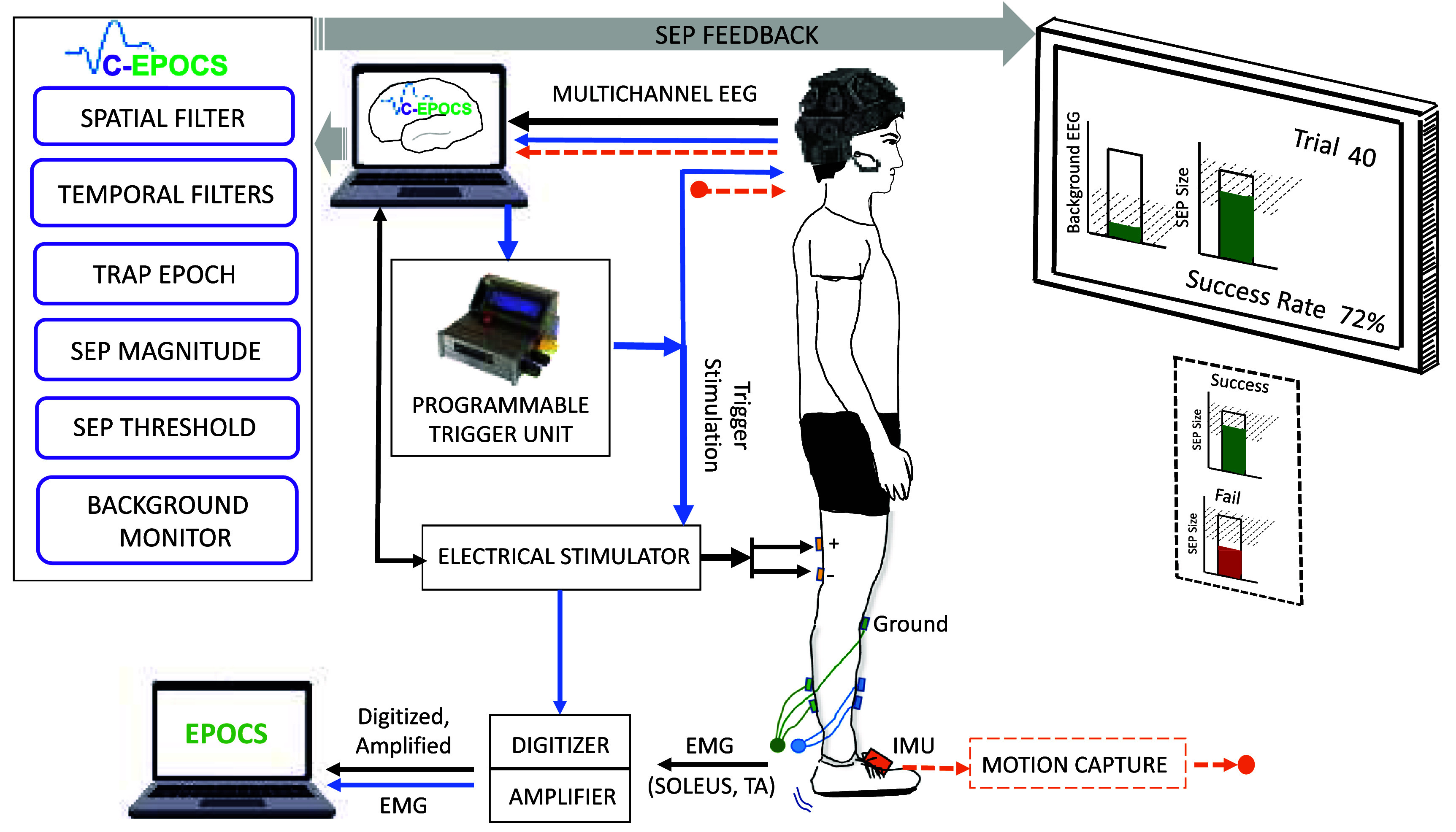
Cortical evoked potential operant conditioning system (C-EPOCS): the C-EPOCS system acquires multichannel EEG data from the headset along with the trigger onset signal. It also monitors the background EEG to check that it remains at a predefined low level, before triggering the electrical stimulator. The background activity is rendered as a gauge on the display monitor. The stimulator stimulates the tibial nerve and evokes an SEP response. This is processed in real time by the C-EPOCS processor, resulting in a one-dimensional SEP magnitude that is rendered as the height of a gauge on the display. Simultaneously, optionally, EPOCS can be separately deployed to record the amplified and digitized EMG from soleus and tibialis anterior muscles, along with a copy of the trigger onset. Wireless inertial measurement units are placed on the feet to capture foot movement due to the stimulation induced twitch.

*e) Online demonstration:* next, we evaluated this procedure in a real-time setup. This step demonstrates the online SEP extraction using an extended version of the EPOCS system referred to as the Cortical-EPOCS (figure 2). The EEG was acquired in the same way as described above, using the DSI-24 headset, and the peripheral stimulation was also applied in the same manner as described above. Primarily, the real time system processed the EEG data in real time, to extract single-trial SEPs, and delivered visual feedback of its magnitude on a bar gauge within approximately 200 msec with respect to stimulation onset. This system was tested with one participant on a separate day from the calibration data collection day described above. The recruitment curve was obtained on the day of this test, as outlined earlier, to maintain consistent afferent excitation across sessions.

*Cortical-EPOCS system:* the EPOCS architecture involved the transmission of analog EMG signals that are digitized independently by an analog-to-digital convertor (such as the PCIe-6321, NI. The interface with the NI unit was also used to drive the electrical stimulator. The EMG signals were processed in real-time and trigger the electrical stimulator when the background EMG meets a specific criterion, such as being maintained at a predefined low level for a predetermined duration. In contrast, an operant conditioning system driven by cortical signals is required to support commercial-grade EEG acquisition systems that typically integrate the pre-amplifiers, amplifier, and digitizer into a single unit, and transmit digitized EEG signals. In the absence of the NI interface, an alternate method is also required to interface with and drive the electrical stimulator with precise timing. Moreover, EEG systems record multiple channels (as many as 32, 64 or even 256) at sampling rates of 300–1200 Hz, with temporal and spatial filtering in real time, with the trigger criterion alternatively based on maintaining a pre-defined low background noise level. To address these challenges, the following main hardware, and software customizations were made:

(i) *Source Module:* EPOCS software was adapted to receive the digitized multichannel data from the EEG headset instead of the NI digitizer as used in EMG-based EPOCS, via an updated BCI2000 source module dedicated to seamlessly interface with this headset.
(ii) *Programmable Trigger unit:* In the EPOCS setup, the serial interface with the NI unit allows it to direct the NI unit to generate and transmit a TTL pulse, activating the electrical stimulator. As a replacement, we designed and used a programmable event synchronization embedded system (Sync Genie (Alkhoury *et al* 2024)) using a microcontroller board with the ARM Cortex-M7 processor. It receives a command from C-EPOCS, generate an instantaneous TTL pulse, and transmit the TTL pulse via digital BNC output ports.
(iii) *SerialInterface Extension Library:* Next, a software extension to BCI2000 (Schalk *et al* 2004) was developed to allow the C-EPOCS to flexibly communicate to and from serial-port devices such as the programmable event synchronization embedded-system. This extension has been publicly released as a contribution to the larger open-source BCI2000 platform maintained by National Center for Adaptive Neurotechnologies (NY). On the embedded-system side, a general-purpose serial-port communication and debugging library, Keyhole (Hill 2023), was developed to facilitate and troubleshoot the interaction. This has also been released to the public domain as part of the Arduino ecosystem. The documentation for these software libraries is available at the respective wiki pages at the BCI2000 web portal. Additional software libraries (SignalAquisition (Hill *et al* 2023), SerialWidgetADC (Hill *et al* 2023)) were also developed and released, used for the development of the embedded system and for protocol testing.

## 3. Statistical analysis

All statistical analysis was performed with MATLAB 202b (Mathworks, MA). As most data was not normal (Lilliefors test (Lilliefors 1967), *p* > 0.05), non-parametric tests were used. Repeated measures assessments were performed with Friedman Repeated measures test, followed by *post-hoc* tests with Tukey’s multiple comparison procedure. The effect size was estimated with coefficient of concordance (Kendall’s $w$ (Tomczak and Tomczak 2014)) with the equation: $w = \,\frac{{{\chi ^2}}}{{n\left( {k - 1} \right)}}$, where ${\chi ^2}$ is the Friedman test statistic, $n$ is the sample size and $k$ is the number of repeated measurements. The interpretation of Kendall’s $w$ was based on Cohen’s interpretation guidelines (Cohen 1977) of 0.1—< 0.3 (small effect), 0.3—< 0.5 (moderate effect), and ⩾0.5 (large effect). The coefficient-of-determination (*r*^2^) is used to assess the signal-to-noise ratio across trials. In case of assessing and visualizing the spatial SNR (across all electrodes), we use the Bonferroni corrected (Shaffer 1995) -log_10_(*p*/electrodes) for visualization. Classification performance was evaluated with ROC -basedAUC scores. These scores range from 0 to 1, with 0.5 being a random guess and 1 being perfect classification (Bradley 1997).

## 4. Results

Data from eleven participants (5 men/6 women; mean age: 45.7 ± 19.7 years; mean height: 65.8 ± 4.0 inches) was analyzed. SEP N70 was observed in all participants, at the central scalp region. The M-wave size was 14.4% ± 12.3% of the *M*_max_ on average, while the background EMG was maintained at 1.4% ± 0.05% of *M*_max_ on average. The average absolute stimulation intensity across participants and runs was 10.5 ± 0.6 mA. In a previous study analysis, we had observed that the SEP N70 acquired during an HrTNP protocol, attenuates significantly with increase in stimulation frequency. Here, we examine whether this attenuation in amplitude affects the classification performance of the N70, particularly for use in real-time decoding applications. We also evaluate this single-trial decoding classification performance in two individuals with CNS injuries (2 men; mean age: 65.5 years)—an individual with incomplete cervical spinal cord injury, and another individual with chronic stroke.

## 5. Feature selection

The recruitment curve data for each participant, and at each stimulation frequency, was first analyzed to identify the spatial and temporal characteristics of the SEP N70 per participant, for subsequent analysis. The average latency of N70 was found to be 98.6 ± 6.5 ms, 86.6 ± 2.3 ms, and 92.9 ± 6.3 ms, at 0.2 Hz, 1 Hz and 2 Hz respectively.

The spatial regions of interest included the central scalp region (Cz electrode), along with neighboring regions known to be involved in sensorimotor responses (i.e. C3, C4, and P3, Pz, P4). These electrodes were used in multivariate decoding analysis. For univariate analysis, we assessed restricting the region-of-interest to the Cz electrode. This selection was formed based on literature and prior studies. It was further supported by the *r*^2^ analysis of the recruitment curve data, which consistently showed a relatively larger *r*^2^ for N70 at Cz compared to the above electrode set. This pattern was observed at all stimulation frequencies, with an *r*^2^ of 0.4 ± 0.2 at 0.2 Hz, 0.2 ± 0.1 at 1 Hz, and 0.3 ± 0.2 at 2 Hz, indicating an appreciable signal-to-noise ratio at Cz. In one participant, the *r*^2^ at 2 Hz frequency for the recruitment curve data was quite poor at all electrodes, and Cz was selected based on their data at lower frequencies. Furthermore, the multivariate analysis also consistently showed a relatively larger contribution of the Cz electrode in the rLDA model, compared to the neighboring electrodes (C3, P3, C4, P4, and Pz).

## 6. Offline classifier calibration

**Figure 3.**
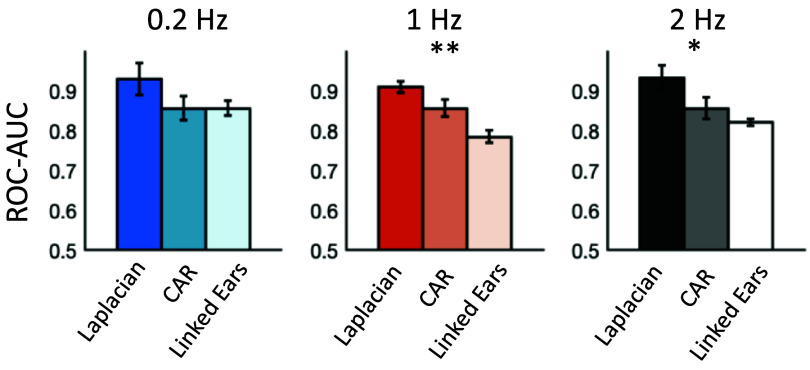
Multivariate offline calibration assessment with linear discriminant analysis, at three stimulation frequencies and with various levels of spatial filtering. Receiver operating characteristics-area under the curve (ROC-AUC) scores are used to measure classification performance. Friedman test for repeated measures shows a significant effect of spatial filter at the higher stimulation frequencies (*p* = 0.003 at 1 Hz and *p* = 0.013 at 2 Hz).

The offline calibration phase was evaluated with a rLDA and 5-fold cross-validation, using AUC as the performance metric. Multivariate offline calibration was evaluated at the three stimulation frequencies—0.2 Hz, 1 Hz and 2 Hz–using three spatial filtering levels: laplacian filtering, common average referencing, and no additional filtering (i.e. using the linked-ears reference montage applied during EEG recording). The N70 AUCs are tabulated in table 1 and shown in figure 3. The classification performance was observed to be highest with a Laplacian filter, across all stimulation frequencies, and relatively lower with a common average reference or a linked ears reference. The difference due to the spatial filter was most prominent at 1 Hz (Friedman Test: ${\chi ^2}$= 11.4, *p* = 0.003, *w* = 0.57), and 2 Hz (${\chi ^2}$= 8.62, *p* = 0.013, *w*= 0.39), with *post hoc* tests showing a significant difference between Laplacian and CAR filters (*p* = 0.002 for 1 Hz, and *p* = 0.013 at 2 Hz).

**Table 1.** Receiver operating characteristics-area under the curve (ROC-AUC) scores (median + standard error, across participants) for multivariate and univariate offline calibration at three stimulation frequencies, at various levels of spatial filtering.

**Multivariate**	**0.2 Hz**	**1 Hz**	**2 Hz**
Laplacian	0.93 ± 0.04	0.91 ± 0.01	0.93 ± 0.03
CAR	0.86 ± 0.03	0.86 ± 0.02	0.86 ± 0.03
Linked ears	0.86 ± 0.02	0.78 ± 0.01	0.82 ± 0.01

**Univariate**	**0.2 Hz**	**1 Hz**	**2 Hz**

Laplacian	0.89 ± 0.03	0.82 ± 0.01	0.82 ± 0.02
CAR	0.86 ± 0.02	0.81 ± 0.02	0.80 ± 0.02
Bipolar	0.82 ± 0.03	0.76 ± 0.02	0.82 ± 0.02

**Figure 4.**
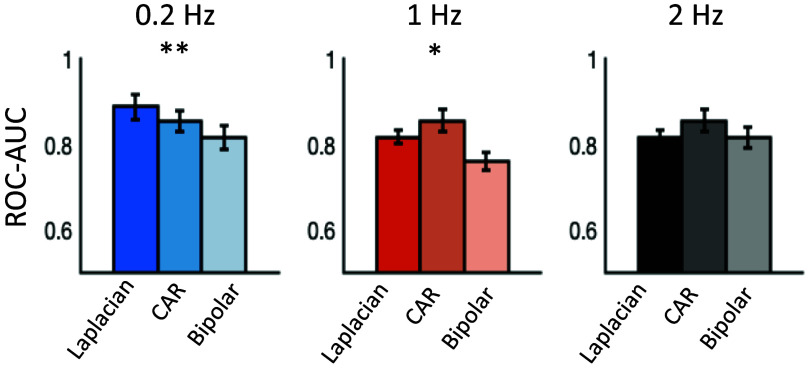
Univariate offline assessment of SEP N70 at three stimulation frequencies, and using three spatial filter options: laplacian, common average reference (CAR) and bipolar. friedman test for multiple comparisons shows a significant effect of filter type at 0.2 Hz (*p* = 0.008) and 1 Hz (*p* = 0.03).

The univariate offline calibration was evaluated at the three stimulation frequencies as well, with a Laplacian filter, CAR filter and a bipolar montage. AUC scores are tabulated in table 1 and shown in figure 4. A significant effect of filter type was observed at 0.2 Hz (Friedman test, ${\chi ^2}$= 9.77, *p* = 0.008, *w*= 0.5), with *post hoc* tests showing a significant difference between Laplacian and bipolar (*p* = 0.01), and CAR and bipolar filters (*p* = 0.03). A significant effect of filter type was also observed at 1 Hz (${\chi ^2}$= 7.26, *p* = 0.03, *w*= 0.36), with *post hoc* tests showing a significance between Laplacian and bipolar filter (*p* = 0.04).

The Laplacian spatial filter performed relatively better than other spatial filtering options in multivariate analysis, and it performed better or equivalent to CAR in univariate analysis. It also showed consistent performance across the three stimulation frequencies (multivariate: ${\chi ^2}$= 3.00, *p* = 0.22, *w* = 0.15; univariate: ${\chi ^2}$= 4.5, *p* = 0.10, *w* = 0.22). Based on these observations, we opted to use Laplacian filter for further analysis.

**Figure 5.**
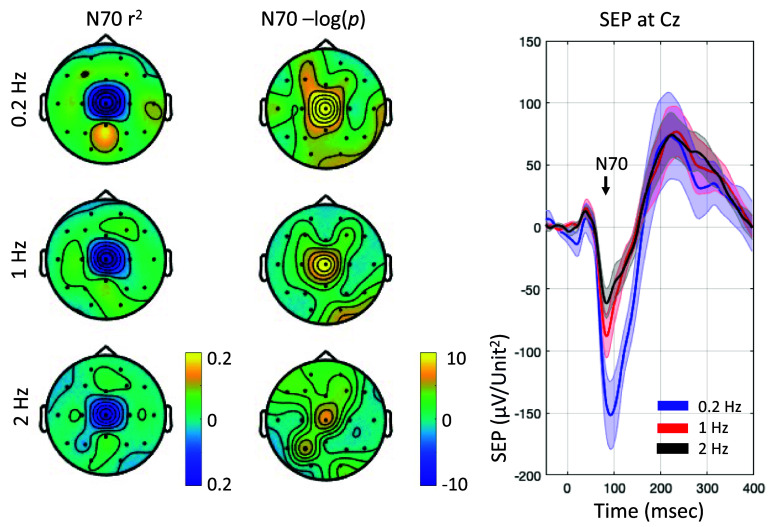
Scalp topographies for offline calibration data at the three stimulation frequencies. Left panel shows the coefficient of determination (*r*^2^) for the somatosensory evoked potential component N70 at all electrodes across all participants. Right panel shows the Bonferroni corrected significance value of these *r*^2^s.

Next, we retrospectively examine the signal-to-noise ratio of the task-related response in this calibration dataset, and its specificity at the central electrodes. We perform this by calculating the *r*^2^ at each electrode and visualizing it as a scalp topography (shown in figure 5, left column). The central scalp region was found to have the largest and most significant *r*^2^ for an SEP N70 response. The significance values across electrodes are visualized as -log_10_(*p*) for ease of visual interpretation (figure 5, middle column). The mean -log_10_(*p*) across participants was larger than the Bonferroni corrected value of 2.6 at all frequencies. The SEP morphology at the Cz electrode showed a prominent N70 component.

## 7. Pseudo-online classification generalization

Next, we test the performance of the decoding generalization by applying the rLDA model created in the calibration phase to predict the SEP N70 from the unseen second run of each stimulation frequency (i.e 75 trials per participant). This dataset was minimally pre-processed, as it would be by an online BCI system, hence referred to as pseudo-online, with the application of only the linear (0.2–40 Hz band pass) and spatial filter. Laplacian spatial filter was used in this analysis, based on the filter selection in the calibration phase.

**Figure 6.**
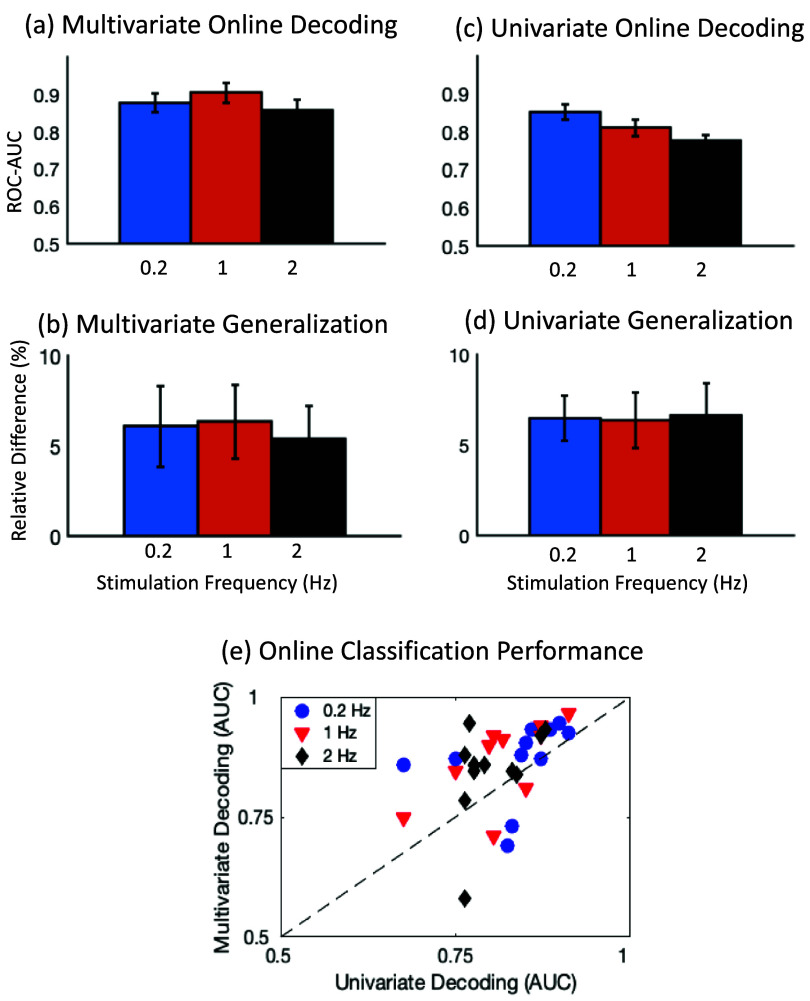
Online decoding performance for multivariate and univariate analysis, across three stimulation frequencies. (a) Multivariate online classification area under the curve (AUC) scores at 0.2 Hz, 1 Hz and 2 Hz. (b) Relative difference in multivariate classification performance from offline to online decoding. (c) Univariate online classification AUC scores at 0.2 Hz, 1 Hz and 2 Hz. (d) Relative difference in univariate classification from offline to online decoding. (e) Comparison of multivariate and univariate online classification performance. Three symbols denote the performance at the three frequencies.

The multivariate classification performance on unseen test data showed AUC scores of 0.88 ± 0.02, 0.90 ± 0.03, and 0.86 ± 0.03 at 0.2 Hz, 1 Hz and 2 Hz respectively (figure 6(a)). The relative difference between offline and pseudo-online decoding performance was similar across the three stimulation frequencies (figure 6(b)): 6.1 ± 2.2% for 0.2 Hz, 6.4 ± 2.0% for 1 Hz, and 5.4 ± 1.8% for 2 Hz, (Friedman Test: ${\chi ^2}$= 1.1, *p* = 0.58, *w* = 0.05).

The univariate pseudo-online detection performance on unseen test data was 0.85 ± 0.02, 0.81 ± 0.02, 0.78 ± 0.01, for 0.2 Hz, 1 Hz and 2 Hz respectively (figure 6(c)). The relative difference from offline to pseudo-online decoding was 6.5 ± 1.3%, 6.4 ± 1.5% and 6.6 ± 1.7% for 0.2 Hz, 1 Hz and 2 Hz respectively, which was expected. These differences were also similar across the stimulation frequencies (${\chi ^2}$= 2.92, *p* = 0.23, *w* = 0.15) (figure 6(d)).

To compare multivariate and univariate classification performance, we plot their AUC scores against each other (figure 6(e)). It illustrates that multivariate classification generally performs better in the pseudo-online decoding at the three stimulation frequencies. Nevertheless, the univariate performance is also reasonably high (>0.75) at these stimulation frequencies.

## 8. Single-trial SEP measurement in spinal cord injury: pilot study

We evaluated the tibial nerve stimulation-based single-trial SEP measurement in a participant with incomplete cervical spinal cord injury. He had tetraplegia and relied on a wheelchair for mobility. The setup was the same as in the rest of the study, with HrTNP stimulation parameters. A stimulation frequency of 1 Hz was chosen as it offered a reasonable trade-off between session duration and signal quality. The data pre-processing pipeline was identical to that used for healthy controls, with the Laplacian filter selected as the spatial filter.

*Multivariate classification* with rLDA showed an AUC score of 0.86, that was 5.6% lower than the median multivariate AUC score (0.91 ± 0.01) at 1 Hz in healthy people.

For univariate *analysis*, the recruitment curve data was initially used to identify the electrodes showing the most significant N70 response and to estimate the N70 latency. The r^2^ for the Cz electrode was found to be largest (i.e. 0.17) and the only significant response at *p* < 0.05 after Bonferroni correction (i.e. a -log(*p*) of 8.24). The latency of the N70 was found to be 95.0 ms, which was 9.7% slower than the mean N70 latency (86.6 ± 2.3 ms) found in healthy people. These features were used in the univariate single-trial SEP measurement, and offline classification performance was assessed with 5-fold cross-validation. N70 could be classified from background EEG with an AUC score of 0.68, 16.05% lower than the median AUC score (0.81 ± 0.07) observed at 1 Hz stimulation frequency in healthy people.

**Figure 7.**
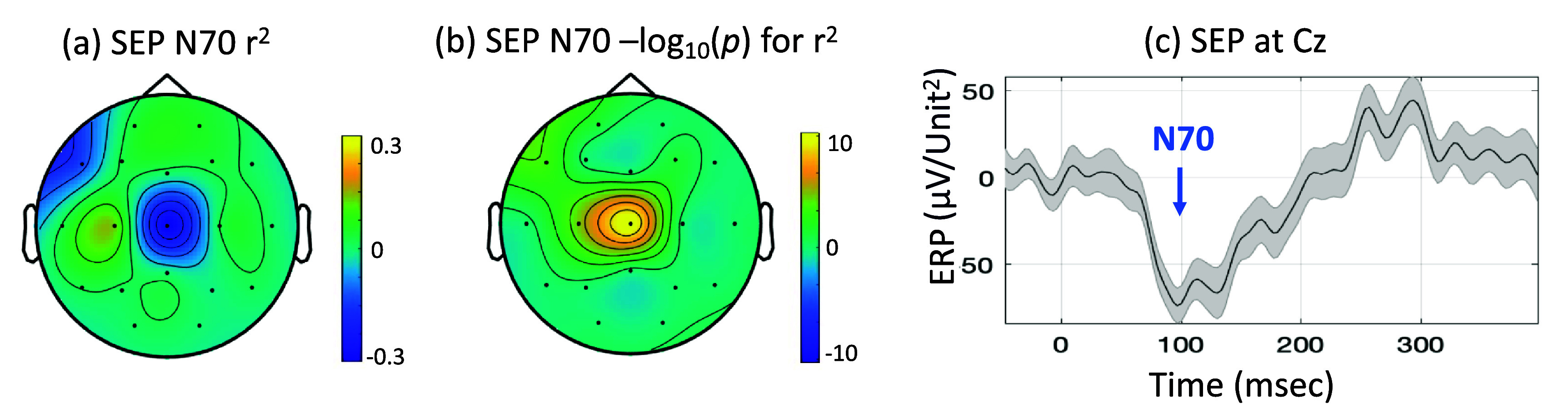
Somatosensory evoked potential (SEP) assessment within an HrTNP setup in an individual with incomplete cervical spinal cord injury (tetraplegia). (a) Scalp topography displays the distribution of the coefficient of determination (*r*^2^) for the SEP component N70 seen to be localized at the central scalp region at Cz electrode. (b) The significance of r^2^ is plotted as the -log10 (p) across the scalp and shows spatial specificity at Cz. (c) The SEP evoked response for tibial nerve stimulation is shown. The signal is noisier than healthy controls, but an N70 component is distinguishable.

Next, we examined the N70 component from the calibration data to confirm its spatial localization (shown in figure 7(a)). We found it consistent with observations in healthy people (shown in figure 5). The *r*^2^ across the electrodes demonstrated spatial specificity at the central scalp regions, with Cz showing the largest and most significant *r*^2^ at *p* < 0.05 (figure 7(b)). The *r*^2^ value at Cz was also a reasonably high value of 0.37, indicating a distinct N70 response relative to the background fluctuations.

The morphology of the averaged SEP (figure 7(c)) appeared visually similar to that of healthy controls (shown in figure 5), albeit noisier, potentially due to a greater trial-to-trial variation or a contamination from power wheelchair motor induced electrical noise. However, it still displayed a discernible N70 component in the averaged SEP signal and was classifiable. The amplitude of N70 was observed to be smaller (−71.4 *μ*V/unit^2^, compared to −88.3 *μ*V/unit^2^ in healthy people), however this data had much more noise contamination, and the stimulation induced twitch was rather small, which would gate the SEP less than that in healthy people at the same stimulation frequency.

## 9. Single-trial SEP measurement in stroke: pilot study

The SEP single-trial measurement was also assessed in one participant with chronic stroke who was participating in a concurrent HrTNP SEP evaluation. The data acquisition and the tibial nerve electrical stimulation setup was the same as described in the Methods section. The stimulation frequency of 1 Hz was being used for the HrTNP protocol, which was also appropriate for the SEP acquisition. The data-preprocessing pipeline was the same as that used for single-trial SEP decoding in healthy controls, based on Laplacian spatial filter.

For single-trial analysis multivariate offline classification showed an AUC score of 0.81, 11.0% lower than the multivariate AUC score observed in healthy controls at 1 Hz (0.91 ± 0.01).

Features for univariate analysis were selected with the help of recruitment curve data—to identify the electrode with the most significant N70 activation for tibial nerve stimulation following a stroke, and to estimate the N70 latency. The Cz electrode was found to have the largest r^2^ value for the N70 (i.e. 0.13), consistent with that observed in healthy controls. Cz *r*^2^ was also the most significant at *p* < 0.05 (Bonferroni corrected), with a -log_10_(*p*) of 5.4. The N70 latency was observed to be 93.3 ms, which was 7.7% slower than the mean latency of 86.6 ± 2.3 ms observed in healthy controls. Univariate offline classification performance was assessed using the 5-fold cross-validation. The AUC score was found to be 0.80, indicating reasonably good performance, and only 1.2% lower than the univariate AUC score seen in healthy controls at 1 Hz stimulation frequency (0.81 ± 0.07).

**Figure 8.**
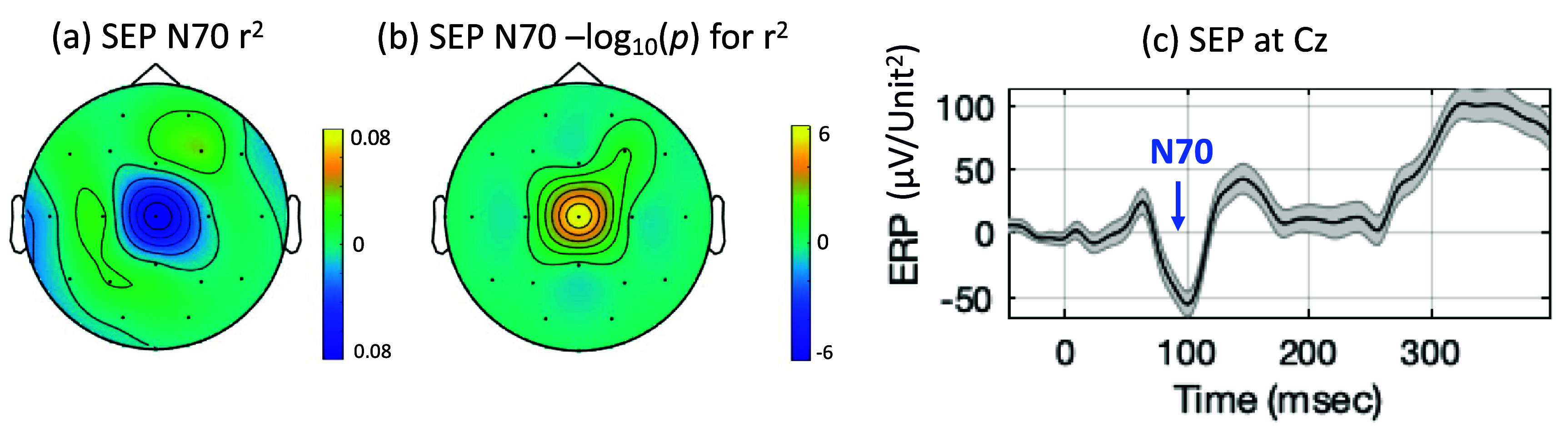
Somatosensory evoked potential (SEP) assessment in a participant with chronic stroke. (a) Scalp topography shows the spatial distribution of the coefficient of determination (*r*^2^) for the SEP component N70. The small value of the *r*^2^ indicates a low SNR (b) Significance of the *r*^2^ for N70 SEP, displayed as Bonferroni corrected -log10 (*p*-value), (c) time course of the SEP at Cz electrode shows a prominent N70 component.

Retrospective analysis of the averaged SEP from this calibration (assessment run) dataset showed that the N70 was indeed most prominent at the central scalp region, similar to its location in healthy controls. The *r*^2^ topography showed specificity of activation at Cz, being the only electrode that showed a significant response at *p* < 0.05 (or -log_10_(*p*) of 6.4) with Bonferroni correction for multiple comparisons. The *r*^2^ topography is shown in figure 8(a) and the corresponding -log_10_(*p*) topography is shown in figure 8(b). The *r*^2^ value indicated an SNR of 0.10, which is more than 50% smaller than the average SNR observed in healthy controls, although the N70 was still classifiable.

The SEP morphology at Cz, as shown in figure 8(c) is similar to that of healthy controls (shown in figure 5), with visible P40 and N70 components. The amplitude of N70 was observed to be smaller, −52.4 *μ*V/unit^2^, compared to −88.3 *μ*V/unit^2^ in healthy controls. We note that the subsequent P200 component was also noticeably depressed or delayed.

## 10. Demonstration of a real time online application

We assessed the feasibility of using the proposed stimulation parameters, while monitoring the afferent input, for extracting individual SEP responses in a real-time setup. First, EPOCS was used to obtain the recruitment curve on the day of the session to determine the stimulation intensity that elicits an *M*-wave that is 10%–20% of *M*_max_ and an *H*-reflex that is ∼75% of *H*_max_. The recruitment curve data was examined there and then using the EPOCS ‘Analysis Module’ to determine the Target stimulation intensity for that session.

Next, C-EPOCS was used to capture the EEG in a realtime mode, where multichannel data was being acquired, temporally filtered (0.2–40 Hz), followed by Laplace spatial filtering, using the built in temporal and spatial filter modules available in the core BCI2000 platform that underlies C-EPOCS. The rms value of the epoched response at Cz (for univariate approach) was obtained. If the resulting response was determined to be larger than background noise, then its magnitude would be displayed as a gauge on the screen within 200 msec. Data analysis shows an AUC score of 0.89, similar to the AUC score (0.86) observed for this participant for the data tested in a pseudo-online setup.

## 11. Discussion

Extraction of single-trial SEPs can be useful for multiple applications, such as a brain computer interface, for rehabilitation. The SEPs measured in real-time in an HrTNP protocol are useful for monitoring the cortical responses along with the peripheral responses, as well as for response feedback. We assessed the offline and online classification performance of SEP N70 within an HrTNP setup. The mid-latency N70 component was examined because of its potential role in the supraspinal sensorimotor feedback loop for lower limbs (Urasaki *et al* 1985). Single-trial non-invasive SEP extraction is being actively studied and various signal processing methods are being developed and tested to meet its real-time extraction challenges. Methods such as Weiner method (Walter 1968), Wavelet filtering (Quiroga and Garcia 2003), multi linear regression (Mayhew *et al* 2006), Bayesian estimation (Truccolo *et al* 2003, Wu 2014), canonical decomposition (Vanderperren *et al* 2013), expectation maximization (Chen *et al* 2016), and Blind source separation (Hu 2005, Rossi 2007, Hu *et al* 2011) have been shown to be useful for assessing single trial responses. However, these methods are retrospective analysis that use information across trials and cross-trial noise information to extract the single trial responses. Also, most of these studies evaluate SEPs elicited with the typical SEP stimulation parameters which differ from those used here (i.e. shorter pulse widths, lower stimulus intensities, faster stimulation frequencies, and without maintenance of stable muscle contraction or stable effective stimulus strength).

Subtle changes in stimulation electrode positions, muscle contraction or temperature are known to affect the effective afferent excitation delivered to the afferent nerves, which can introduce variability across trials and sessions. Maintaining a stable afferent input may therefore help to reduce some of the variability.

### 11.1. Classification performance in healthy participants

In healthy participants, we typically observed a good classification performance across all stimulation frequencies during offline calibration, especially with multivariate classification. The spatial filter (Laplacian/common average/bipolar) showed the most prominent effect at the slower frequencies for univariate analysis, and a minimal difference in performance at the fastest frequency of 2 Hz. This may be explained by the potentially greater and more distributed neural interference at the slower rate, as it involves longer pauses and extended session durations, potentially inducing fatigue, and distraction. A CAR filter helps remove common-mode noise by assuming a globally uniform distribution of noise across all electrodes. However, it is less effective in removing spatially focused noise and may even introduce noise into the electrode of interest if noise is localized in other areas (McFarland *et al* 1997, Luck 2014). In contrast, a Laplacian filter behaves like a high-pass spatial filter that enhances localized activity. It is less sensitive to global noise from distant sources (McFarland *et al* 1997). Similarly, a bipolar filter can reduce local noise and is less affected by noise at distance areas. However, it relies heavily on the choice of the reference electrode and its signal quality, such that if this electrode is noisy, it can degrade the quality of the referenced signal, making the bipolar method less robust to variations across sessions.

On the other hand, in multivariate classification, the spatial filter choice had a larger effect at higher stimulation frequencies of 1 Hz and 2 Hz. The classification performance was better with Laplacian filter and degraded with CAR or linked ears reference. This may be explained by the inherent strength of multivariate analysis, where transient variations at an electrode may be overcome by the combination of information from neighboring electrodes. However, at higher stimulation frequencies, it is possible that there are more perturbations and artifacts even at neighboring electrodes, such as in maintaining balance, that spatial localization with Laplacian or CAR that improve the SNR of the SEP, can improve the classification performance considerably.

The signal to noise ratio of the N70 was found to be largest at the spatially filtered Cz electrode. The multivariate analysis also showed a larger contribution of the Cz electrode. This is expected, as the lower limb stimulation has been previously shown to be most associated with brain activity at the central scalp region (Roux *et al* 2018). This region also follows the sensory homunculus (Avanzini *et al* 2018), and the cytoarchitectural regions associated with lower limb somatosensory processing (i.e. Brodmann areas 1, 2 and 3) (Standring 2021). An increase in stimulation frequency does not appear to affect the spatial specificity of the N70; however, it considerably reduces its signal-to-noise ratio. This degradation impacts classification performance, although not significantly. Its effect on generalizability across sessions remains to be tested.

### 11.2. Pseudo-online generalization performance deteriorates at high frequency

The decoder maintains a reasonably good generalization performance with an AUC score > 0.78 for multivariate and univariate analysis, when applied on unseen minimally-denoised test data. The degradation from offline calibration to online decoding is on average 5.9 ± 0.30% for multivariate classification, and 6.5 ± 0.06% for univariate classification, across the three stimulation frequencies. The online univariate classification performance was observed to be lower at the faster stimulation frequency of 2 Hz, as compared to the slower 0.2 Hz, although not statistically significantly. This may be explained by the multiple variables and sources of noise at higher stimulation frequencies, such as larger foot movement, lesser perceptible variability in inter-stimulus interval, and the decreased signal-to-noise ratio of the N70 itself. The intermediate 1 Hz stimulation frequency offers a reasonable trade-off, providing stable and generalizable decoding (with both multivariate and univariate approaches) while allowing for a relatively shorter experimental session.

### 11.3. Classification of SEPs in incomplete cervical spinal cord injury (SCI)

The single-trial decoding was assessed for tibial nerve stimulation at 1 Hz stimulation frequency in an individual with incomplete cervical SCI. The SEPs were acquired with the HrTNP protocol settings. The SEP N70 was observed at Cz–central scalp region, similar to healthy controls. The SEP was however a noisier signal in general, with an N70 that was slightly delayed (9.7% or 8.4 ms slower), than that in healthy controls. Nevertheless, the offline classification performance achieved an AUC of 0.68, which is 16.05% lower than healthy controls, and higher than chance level. Multivariate classification approach showed a much higher AUC of 0.86, that was 5.6% smaller than healthy controls.

### 11.4. Classification of SEPs in chronic stroke

As the 1 Hz stimulation frequency demonstrated a good SEP extraction and generalizability in healthy people, we evaluate it in a participant with chronic stroke. The SEP N70 was found to be considerably weak. It was also slightly delayed (7.7% or 6.7 ms slower) relative to healthy controls. This is expected as various somatosensory responses are known to be affected in people with stroke (Kovala *et al* 1991, Fierro *et al* 1999, Zhang *et al* 2011). The spatial region—central scalp area (Cz)—associated with the N70 was similar to that observed in healthy controls, albeit with a much smaller (more than 50% smaller) signal-to-noise ratio compared to healthy controls, which is also expected as the N70 amplitude is smaller. Despite the degradation of the SEP response, the N70 remained reasonably classifiable during univariate offline calibration, with a high AUC score of 0.80—only 1.2% lower than that of healthy controls at the same stimulation frequency. This is possible if the N70 remains a distinct deflection while the background fluctuations remain minimal. The multivariate classification approach improved it only marginally to 0.81, which was 11.0% smaller than the multivariate AUC score seen in healthy controls. This may indicate noisier neighboring spatial regions, suggesting univariate approach as a preferable approach in such cortical injuries. Although this needs to be evaluated on a larger cohort.

### 11.5. Realtime demonstration of single-trial SEP extraction

The real-time demonstration confirmed the feasibility of online single-trial extraction of SEPs using the proposed stimulation parameters and setup, though further testing is warranted. The results show that this setup can reliably elicit stable, detectable SEPs with an AUC of 0.89—even with a univariate approach with minimal signal processing. Additional signal processing approaches may improve this detectability further and will need evaluation and optimization with regards to the constrained time and information availability during the single-trial processing pipeline. This capability is particularly valuable for real-time feedback in operant conditioning across sessions in individuals with brain or spinal cord injuries. By enabling real-time detection and minimizing the number of discarded responses, it can help sustain participant attention and amplify the impact of reinforcement. Discarded trials may result from attenuated or absent responses caused by habituation, movement, attention lapses, neural or electrical artifacts, electrode malfunctions, or inherent variability in response latency and spatial distribution.

### 11.6. Future applications for SEP based neurorehabilitation

These results highlight the potential of utilizing SEP event related responses in a real time brain computer interface-based application, that essentially requires single-trial decoding. SEP feedback-based conditioning could be useful for augmenting a weakened SEP response, typically observed after brain and spinal injuries. This may help improve the impaired sensory connectivity, indirectly aiding movement rehabilitation by repairing the sensory feedback loop, essential for movement.

## 12. Conclusion

Results showed an excellent offline classification performance for SEP N70 when tested with the HrTNP settings for tibial nerve stimulation at 0.2 Hz, with a small decrease in accuracy at higher stimulation frequencies. The decrease may be due to the attenuated SEP N70 amplitude and SNR at higher frequencies, previously attributed to the gating effect. Generalization performance showed an expected degradation, at all frequencies, with an average decrease of 5.9% (multivariate) and 6.5% (univariate), with an AUC score that ranged from 0.78–0.90. Most importantly, all three frequencies appear to be able to support single-trial SEP decoding and may be usable for an SEP-based brain computer interface.

The pilot assessments of offline single-trial SEP decoding performance in individuals with incomplete cervical spinal cord injury and stroke, demonstrated reasonable classification accuracy, with an AUC of 0.68 (univariate) and 0.86 (multivariate) in iSCI, and 0.80 (univariate) and 0.81 (multivariate) in stroke, despite a weakened N70 response.

The realtime demonstration showed the feasibility of the procedures to elicit detectable SEPs with an AUC of 0.89, a reasonably good score considering the minimal post-processing. Additional signal processing may improve this detectability further and remains to be evaluated.

These preliminary findings and methods highlight the potential of extracting single-trial SEPs for use in brain-computer interface-based training, such as delivering real-time neurofeedback using the SEP magnitude, while minimizing inter-session variability. When integrated with operant conditioning, this approach could be useful in actively and effectively engaging the somatosensory network, facilitating a task-related sensory training—from peripheral sensation perception to visualization of the corresponding cortical response, and being rewarded for enhanced perception. In essence, it may primarily enhance sensory attentiveness to peripheral stimulation, but by doing so, it could lead to greater neurophysiological and sensory-motor rehabilitation benefits than peripheral stimulation alone.

Future development could involve adapting the system to adjust feedback or stimulation based on the participant’s performance, thereby enhancing learning and promoting neural plasticity. Incorporating gamification could also help increase engagement and help prevent habituation. Overall, such R&D studies would help evaluate the synergy between sensory feedback and motor training, offering valuable insights into more effective rehabilitation strategies.

## Data Availability

The data cannot be made publicly available upon publication due to legal restrictions preventing unrestricted public distribution. The data that support the findings of this study are available upon reasonable request from the authors.
